# Efficacy of the visual cognitive assessment test for mild cognitive impairment/mild dementia diagnosis: a meta-analysis

**DOI:** 10.3389/fpubh.2023.1293710

**Published:** 2023-10-31

**Authors:** Jui-Hung Hsu, Chien-Cheng Liu, I-Wen Chen, Jheng-Yan Wu, Po-Yu Huang, Ting-Hui Liu, Kuo-Chuan Hung

**Affiliations:** ^1^Department of Anesthesiology, Chi Mei Medical Center, Liouying, Tainan, Taiwan; ^2^Department of Anesthesiology, E-Da Hospital, I-Shou University, Kaohsiung, Taiwan; ^3^Department of Nursing, College of Medicine, I-Shou University, Kaohsiung, Taiwan; ^4^School of Medicine, I-Shou University, Kaohsiung, Taiwan; ^5^Department of Nutrition, Chi Mei Medical Center, Tainan, Taiwan; ^6^Department of Internal Medicine, Chi Mei Medical Center, Tainan, Taiwan; ^7^Department of Psychiatry, Chi Mei Medical Center, Tainan, Taiwan; ^8^Department of Anesthesiology, Chi Mei Medical Center, Tainan, Taiwan; ^9^School of Medicine, College of Medicine, National Sun Yat-sen University, Kaohsiung, Taiwan

**Keywords:** mild cognitive impairment, visual cognitive assessment test, sensitivity, specificity, mild dementia

## Abstract

**Background:**

Mild cognitive impairment (MCI) is an intermediate stage between normal ageing and dementia. The early identification of MCI is important for timely intervention. The visual cognitive assessment test (VCAT) is a brief language-neutral screening tool for detecting MCI/mild dementia. This meta-analysis evaluated the diagnostic efficacy of the VCAT for MCI/mild dementia.

**Methods:**

Medline, Embase, Google Scholar, and Cochrane Library were searched from their inception until August 2023 to identify studies using VCAT to diagnose MCI/mild dementia. The primary outcome was to assess the diagnostic accuracy of the VCAT for detecting MCI/mild dementia through area under the receiver operating characteristic curve (AU-ROC) analysis. The secondary outcome was to explore the correlation between VCAT scores and MCI/mild dementia presence by comparing scores among patients with and without MCI/mild dementia. Pooled sensitivity, specificity, and area under the curve (AUC) were calculated.

**Results:**

Five studies with 1,446 older adults (mean age 64–68.3 years) were included. The percentage of participants with MCI/mild dementia versus controls ranged from 16.5% to 87% across studies. All studies were conducted in Asian populations, mostly Chinese, in Singapore and Malaysia. The pooled sensitivity was 80% [95% confidence interval (CI) 68%–88%] and the specificity was 75% (95% CI 68%–80%). The AU-ROCC was 0.77 (95% CI 0.73–0.81). Patients with MCI/mild dementia had lower VCAT scores than the controls (mean difference −6.85 points, *p* < 0.00001).

**Conclusion:**

VCAT demonstrated acceptable diagnostic accuracy in distinguishing MCI/mild dementia in cognitively normal older adults. As a language-neutral and culturally unbiased tool, the VCAT shows promise in detecting MCI/mild dementia. Further studies in non-Asian populations are required.

**Systematic review registration:**

https://www.crd.york.ac.uk/prospero/, identifier: CRD42023453453.

## 1. Introduction

Within the continuum of cognitive aging, mild cognitive impairment (MCI) represents the intermediate stage between normal aging and dementia (1, 2). A comprehensive review showed that 15.56% of community-dwelling adults aged ≥50 years have MCI (3), and this prevalence rate increases with age, reaching up to 25.2% among those aged 80–84 years (3, 4). MCI frequently manifests as objective cognitive impairments, including memory, thinking, and behavioral deterioration (5). Although MCI does not significantly impair older adults’ daily activities and independence (5), approximately 40% of individuals with MCI eventually develop dementia (6), highlighting that MCI is not a benign condition but rather often a transitional stage to more severe forms of cognitive impairment, including dementia. Compared with MCI, in which older adults’ daily activities and independence are not significantly impaired, mild dementia is characterized by more pronounced cognitive impairments that significantly interfere with daily functioning. Evidence suggests that both MCI and mild dementia are linked to higher mortality rates, suggesting that even minor cognitive deterioration reduces life expectancy (7, 8). A recent large-scale study examined Medicare claims records from 2015 to 2019, covering 38–41 million beneficiaries aged 65 years or older, to ascertain the real-world diagnosis rates for MCI and dementia (9). Findings from 2017 to 2019 showed that only 7.9% of anticipated MCI cases were actually diagnosed (9), implying that approximately 7.4 million cases went undetected. Consequently, considering the vast potential of the cognitively impaired population, there is an increasing need for early diagnosis of cognitive impairment and timely interventions.

Cognitive screening tools, including the Mini-Mental State Examination (MMSE) and the Montreal Cognitive Assessment (MoCA), are commonly used (10, 11). Although the MMSE and MoCA have good diagnostic utility, their clinical application may be limited in detecting MCI across diverse linguistic, cultural, and educational backgrounds (12, 13). The visual cognitive assessment test (VCAT) is a brief language-neutral cognitive screening tool designed to detect MCI/mild dementia. It consists of 10 subtests assessing memory, visuospatial function, and executive function using non-verbal, visually based items without complex verbal instructions (14). The test takes approximately 15–20 min to complete (14). The visual-based format of the VCAT makes it especially appropriate for identifying cognitive impairments in diverse, multicultural, and multilingual populations globally (12). Previous studies have affirmed the efficacy of the VCAT in identifying MCI/mild dementia (12, 15); however, the limited sample size in these studies underscores the need for a systematic evaluation of its effectiveness. This meta-analysis aimed to investigate the efficacy of the VCAT in the diagnosis of patients with MCI/mild dementia.

## 2. Materials and methods

### 2.1. Protocol registration

This review was designed and reported in accordance with PRISMA guidelines. Details of the protocol registration are available in PROSPERO (Number: CRD42023453453).

### 2.2. Data source and study screening

Articles were identified through a search of databases, including MEDLINE, Google Scholar, EMBASE, and the Cochrane Library, spanning from their inception to August 11, 2023. The search was performed employing the following keywords: (“cognitive impairment” or “cognitive decline” or “impaired cognition” or “cognitive dysfunction” or “cognitive disorder” or “cognitive disability” or “cognitive deficit”) and “visual cognitive assessment test.” Moreover, to augment the inclusiveness of the literature exploration, controlled vocabulary search terms, including MEDLINE (MeSH) and EMBASE (Emtree), were used. A manual review of the reference lists from the acquired studies was performed to identify additional studies that might be eligible for inclusion. There were no restrictions based on the language in which the studies were published. The search strategy used to scan the MEDLINE database is presented in Table 1.

**Table 1:** Search strategy for medline.

1	(“Cognitive impairment” or “impaired cognition” or “cognitive decline” or “cognitive dysfunction” or “cognitive disability” or “cognitive disorder” or “cognitive deficit”).mp.
2	Exp “cognitive dysfunction”/
3	(Visual cognitive assessment test).mp.
4	(1 or 2) and 3

### 2.3. Criteria for study selection

The criteria for article inclusion were as follows: first, studies that employed the VCAT for diagnosing MCI/mild dementia irrespective of ethnicity were considered. Second, studies that provided data on the sensitivity, specificity, and number of patients afflicted with MCI/mild dementia were considered. A diverse range of study designs (e.g., cohort, case-control, and randomized controlled studies) were deemed eligible for inclusion in this meta-analysis.

Studies falling into any of the following four categories were excluded from the analysis: (1) non-peer-reviewed reports; (2) abstracts, letters, review articles, and case series; (3) studies devoid of information pertaining to MCI/mild dementia or VCAT; and (4) reports for which full-text versions could not be accessed.

### 2.4. Data collection

Two researchers independently collected the following details: study characteristics (e.g., sample size), demographics of the enrolled patients, criteria for the definition of MCI/mild dementia, ethnicity, language, education level, sensitivity and specificity values, area under the curve (AUC) value, and the time required for VCAT. Any disagreements were resolved by a third investigator. Efforts were made to contact the authors of the studies to acquire missing or incomplete data.

### 2.5. Study outcomes

The main objective was to evaluate the diagnostic precision of the VCAT in diagnosing MCI/mild dementia by calculating the area under the receiver operating characteristic curve (AU-ROC). The criteria for defining MCI/mild dementia were derived from those used in individual studies. The secondary objective focused on the relationship between VCAT scores and the presence of MCI/mild dementia by calculating the score differences between patients with and without MCI/mild dementia.

### 2.6. Quality assessment

To evaluate the quality and potential bias of studies assessing the accuracy of diagnostic tests, the Quality Assessment for Diagnostic Accuracy Studies-2 (QUADAS-2) tool was used (16). The QUADAS-2 tool has four domains: patient selection, index test, reference standard, and flow/timing. Independent reviewers assign judgments of “low,” “high,” or “unclear” risk of bias to each signaling question within the domains. The authors engaged in thorough discussions to reach consensus in instances where disagreements or discrepancies emerged. If discrepancies remained unresolved, a third team member was brought in to facilitate the resolution.

### 2.7. Statistical analysis

In this meta-analysis, ROC curve analysis was employed to determine the area under the curve (AUC), offering a comprehensive gauge of diagnostic accuracy. Furthermore, Fagan’s nomogram was applied to estimate the post-test probability of the disease by integrating the pre-test probability with the test likelihood ratios (17). These ratios provide insights into how much a positive or negative test result alters the likelihood of the disease: values between two and five marginally increase probability, five to ten moderately increase probability, and above ten substantially elevate the post-test likelihood compared to the pre-test probability (18). Deek’s funnel plot was used for publication bias assessment. It analyzes the correlation between study size and effect size. To identify whether the findings were statistically significant, a significance level of 0.05 was set. Statistical analyses were performed using RevMan software or MIDAS command in Stata 15 (StataCorp LLC., College Station, TX, United States).

## 3. Results

### 3.1. Literature search

**Figure 1:**
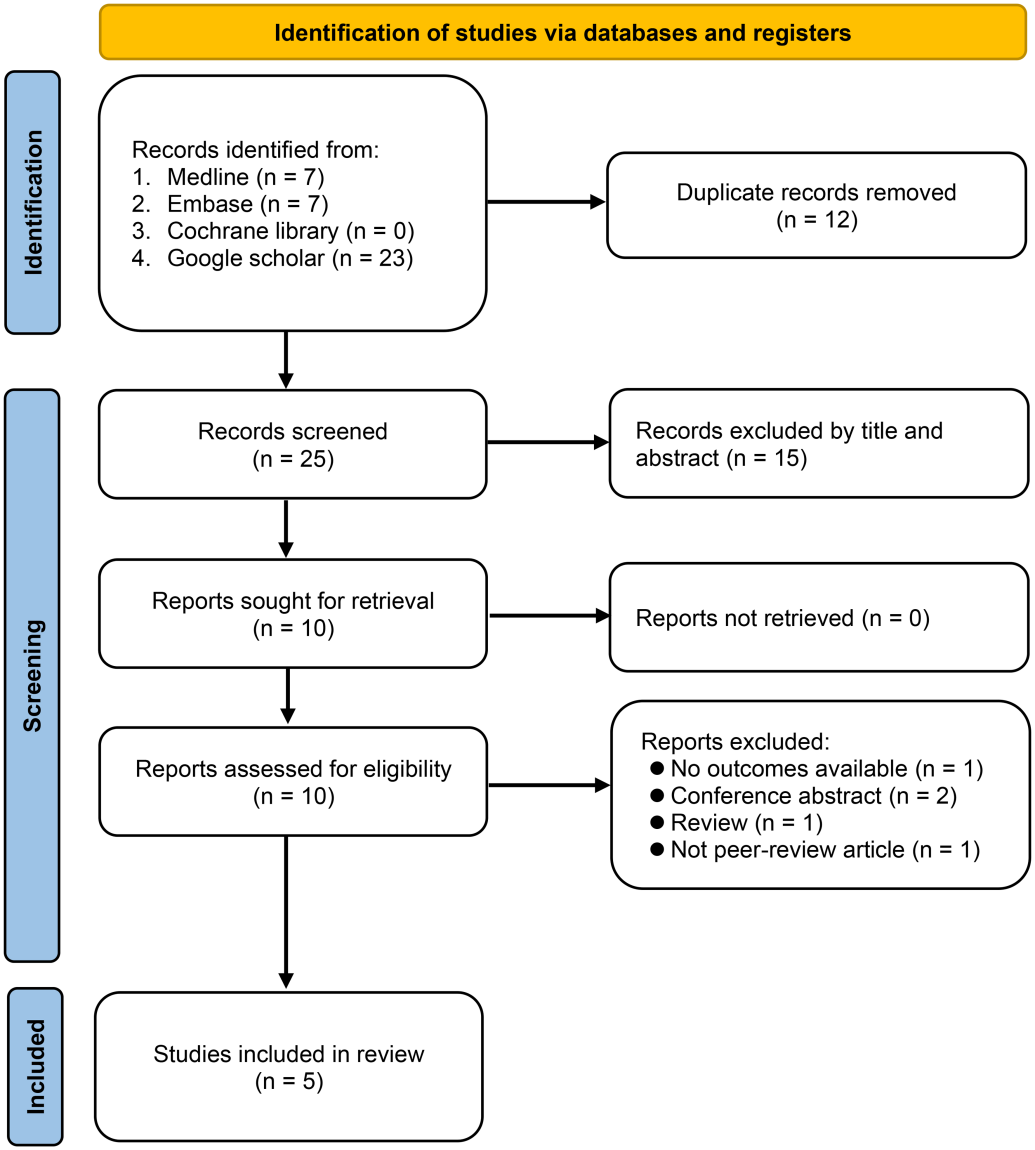
Flow chart for study selection.

The selection process for the eligible studies is shown in Figure 1. The PRISMA flow diagram presented in Figure 1 was constructed based on the recommendations of a previous study (19). During the search process, we did not limit the publication year. Given that VCAT is a new tool, only a limited number of records were identified. Overall, a comprehensive literature search yielded only 37 records. Of these, 27 were subsequently excluded owing to duplication (*n* = 12) and title- and abstract-based exclusion (*n* = 15). After a thorough full-text reading and assessment of the 10 reports, we decided to exclude 5 of them from our review for various reasons. One study was excluded because it did not provide the necessary outcomes relevant to our review. Two reports were conference abstracts and did not provide comprehensive data for our analysis. Another report was a review, and as our aim was to include the original research, we decided not to incorporate it. Finally, one of the reports was not a peer-reviewed article, and given the importance of peer review in ensuring the quality and validity of research, we deemed it inappropriate for inclusion in our review. Finally, five studies published between 2016 and 2023 were determined eligible for inclusion (12, 15, 20–22). Further search of the reference lists within these selected articles did not reveal any additional studies aligned with the inclusion criteria.

### 3.2. Characteristics of studies and methodological quality

An overview of the study characteristics, collectively comprising 1,446 patients, is provided in Table 2. Notably, all studies encompassed individuals aged >60 years, ranging from 64 to 68.3 years. Regarding sex distribution, a female preponderance was observed, with male proportions varying between 39.5% and 51.4%. The sample sizes varied across the studies, ranging from 184 to 471 participants. Of these, four included patients with MCI or mild dementia of the Alzheimer’s disease (AD) type (12, 15, 20, 21), whereas one exclusively focused on individuals with MCI (22). The percentage of participants with MCI/mild dementia versus controls (i.e., case: control ratio) across these studies ranged from 16.5% to 87%. All the studies included in the analysis were conducted within the geographical confines of Asian countries, with four of them conducted in Singapore (12, 15, 20, 22) and the remaining one in Malaysia (21). The ethnic composition predominantly consists of Chinese individuals, varying between 52.5% and 92.5%. The participants demonstrated an educational background of over 10 years, encompassing durations ranging from 10.5 to 13.59 years. Four studies specified the language employed in assessments (12, 15, 21, 22), while one omitted this detail (20). Three studies reported the time required for VCAT completion (12, 15, 21), indicating that the test did not exceed 20 min.

**Table 2:** Characteristics of studies (*n* = 5).

Study	Mean age (years)	Male (%)	*N*	Healthy control (%)	MCI/MD (%)	Chinese (%)	Mean education level (years)	English administered	Mean time to complete the VCAT (minutes)	Country
Low 2020	64	39.5	471	49.5	MCI: 24.8	92.5	11.01	NA	NA	Singapore
					MD: 25.7					
Lim 2018	67.93	51.4	284	57.7	MCI: 16.5	52.8	11.51	34.5%	10.4 vs. 13.9^a^	Singapore
					MD: 25.7					
Ng 2022	68.8	43.5	184	42.9	MCI: 25	71.7	12	54.9%	10 vs. 15.4^a^	Malaysia
					MD: 32.1					
Kandiah 2016	67.8	46.1	206	35.9	MCI: 19.9	91.7	10.5	70.6%	15.7	Singapore
					MD: 44.2					
Soo 2023	63.64	44.2	301	13	MCI: 87	85.4	13.59	91.7%	NA	Singapore

MCI, mild cognitive impairment; MD, mild dementia; NA, not available.

a Control group vs. MCI/MD group.

**Figure 2:**
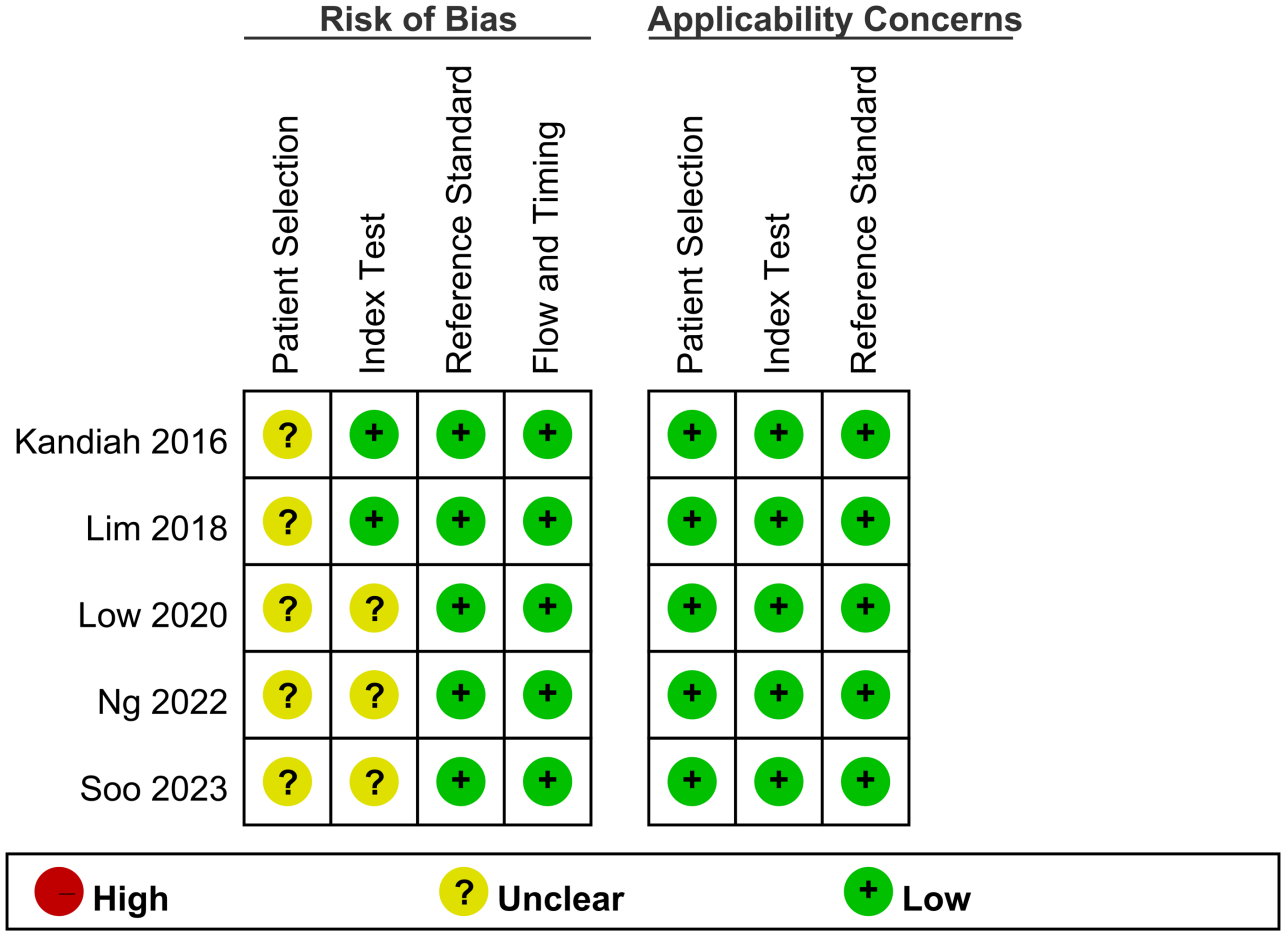
Methodological quality summary.

An overview of the risk of bias and applicability issues in the included studies is shown in Figure 2. One area of concern relates to the selection or recruitment of patients, which could introduce sample bias; consequently, the risk of bias for patient selection remains unclear in all studies (12, 15, 20–22). Regarding the index test, three studies did not pre-specify a threshold for what would constitute a positive test result on the VCAT, leaving the risk of bias in this area also unclear (20–22). All other risks of bias or concerns regarding applicability were deemed low across all the included studies.

### 3.3. Definitions of MCI/mild dementia

The definitions of MCI/mild dementia are summarized in Table 3. For MCI diagnosis, one study adopted the National Institute on Aging and Alzheimer’s Association (NIA-AA) criteria (20), another study employed Petersen’s criteria (22), and three studies concurrently utilized both criteria for diagnosis (12, 15, 21). For mild dementia, four studies relied upon the NIA-AA criteria (12, 15, 20, 21), whereas one study also referenced the National Institute of Neurological Disorders and Stroke and the Association Internationale pour la Recherche et l’Enseignement en Neurosciences criteria (21).

**Table 3:** Definition for mild cognitive impairment and mild dementia.

	Healthy controls	Mild cognitive impairment	mDAD or mVaD
Low 2020	CDR score of 0 without cognitive complaints	CDR score of 0.5 (NIA-AA criteria)	CDR score of 1.0 (NIA-AA criteria)
Lim 2018	MMSE >27 and CRD of 0	≥1 cognitive symptoms; 27 >MMSE >24 with CDR score of 0.5 (Petersen’s criteria)	CDR score of 1.0 (NIA-AA criteria)
Ng 2022	CDR score of 0 without cognitive symptoms	CDR score of 0.5 (Petersen’s criteria and NIA-AA criteria)	mDAD: CDR score of 1.0 (NIA-AA criteria) mVaD: CDR score of 1.0 (NINCDS-AIREN criteria)
Kandiah 2016	MMSE >28 and CRD of 0 without cognitive symptoms	CDR score of 0.5 (Petersen’s criteria and NIA-AA criteria)	CDR score of 1.0 (NIA-AA criteria)
Soo 2023	No cognitive symptoms or functional deficits	Petersen’s criteria and NIA-AA criteria	Patients with mDAD not included

CDR, clinical dementia rating; NIA-AA, National Institute on Aging and Alzheimer’s Association; mDAD, mild dementia of Alzheimer’s disease (AD) type; mVaD, mild vascular dementia; MMSE, Mini-Mental State Examination; NINCDS-AIREN, National Institute of Neurological Disorders and Stroke and Association Internationale pour la Recherche et l’Enseignement en Neurosciences.

### 3.4. Results from individual study and potential limitation

An overview of the diagnostic efficacy of the VCAT within each individual study is presented in Table 4. Notably, four studies exhibited an AU-ROC exceeding 0.8, spanning from 0.837 to 0.933, whereas a single study reported an AU-ROC of 0.794. The sensitivity values ranged from 0.754 to 0.921, whereas the specificity values were within the range of 0.711–0.872. Supplementary Table S1 summarizes the potential limitations enumerated for each study included in the meta-analysis.

**Table 4:** Diagnostic efficacy of visual cognitive assessment test (VCAT).

Study	AUC	Sensitivity (%)	Specificity (%)	PPV (%)	NPV (%)
Low 2020	0.855	75.4	71.1	72.7	73.9
Lim 2018	0.905	92.1	74.2	72.3	92.8
Ng 2022	0.837	77.1	72.2	78.7	70.4
Kandiah 2016	0.933	85.6	81.1	89	76
Soo 2023	0.794	59.5	87.2	96.9	24.3

AUC, area under the curve; PPV, positive predictive value; NPV, negative predictive value.

### 3.5. VCAT values in patients with or without MCI/mild dementia

**Figure 3:**
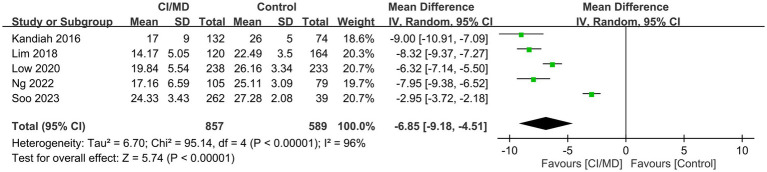
Forest plot showing low visual cognitive assessment test (VCAT) values in patients with mild cognitive impairment (MCI)/mild dementia compared with those without MCI/mild dementia.

Five studies reported VCAT values in patients with (*n* = 857) or without (*n* = 589) MCI/mild dementia. Patients with MCI/mild dementia had lower VCAT values than those without MCI/mild dementia (mean difference, −6.85; 95% CI: −9.18 to −4.51; *p* < 0.00001; *I*^2^ = 96%) (Figure 3) (12, 15, 20–22).

### 3.6. Diagnostic efficacy of VCAT for MCI/mild dementia

**Figure 4:**
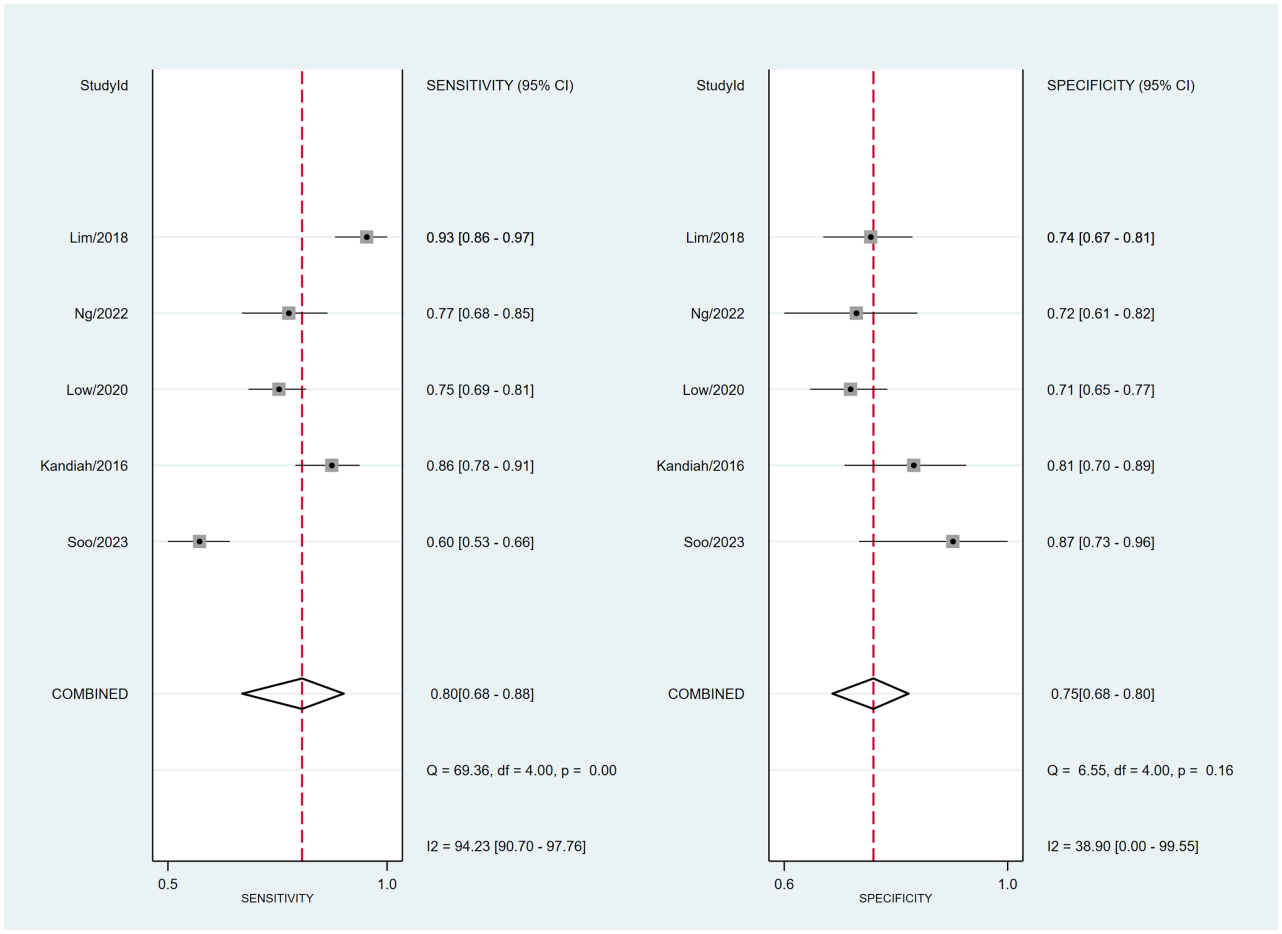
Forest plot depicting the combined sensitivity and specificity of 80% (68%–88%) and 75% (68%–80%), respectively, of the VCAT for mild cognitive impairment (MCI)/mild dementia.

**Figure 5:**
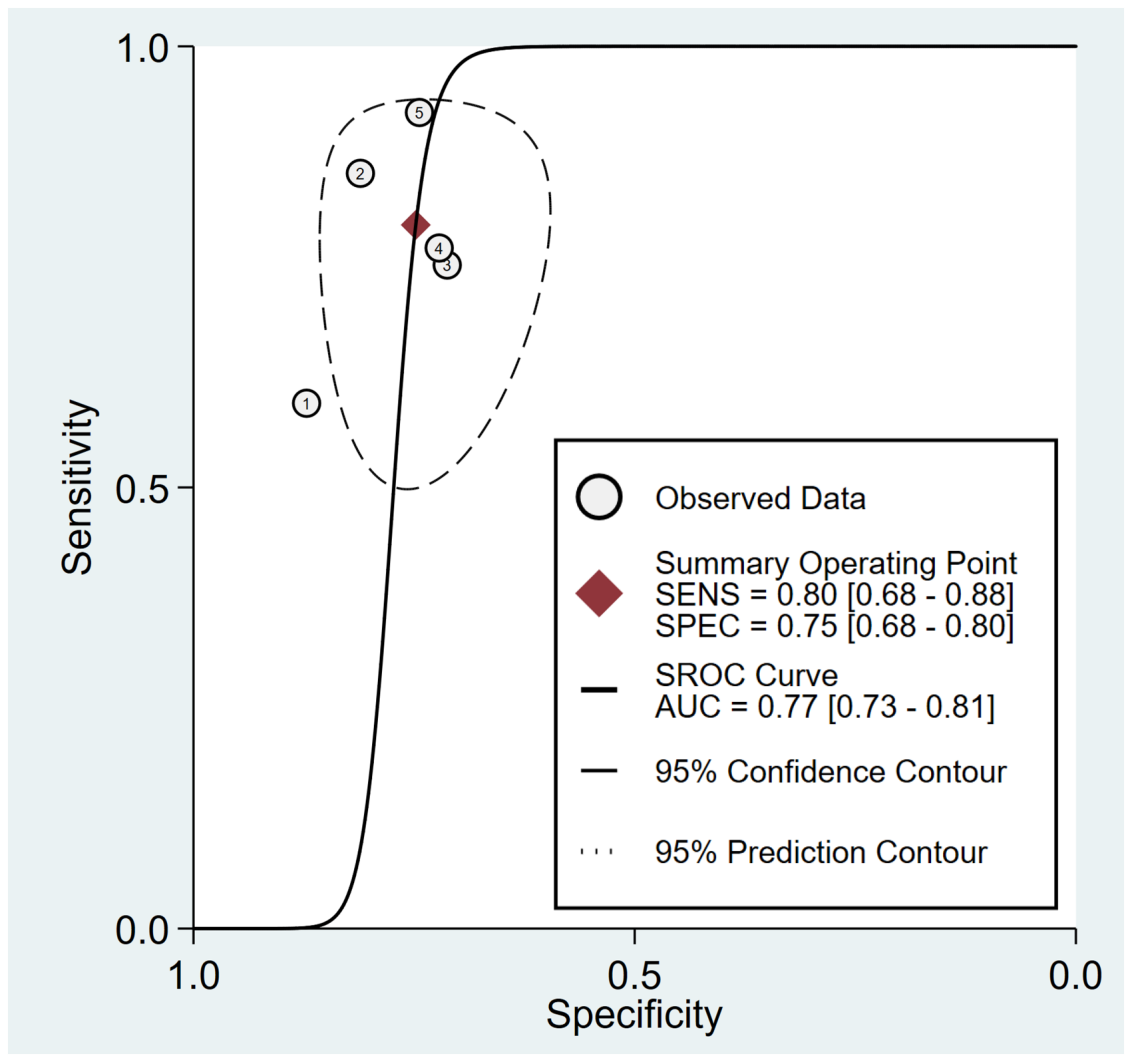
Hierarchical summary receiver operating characteristic (HSROC) curves illustrating an area under the curve (AUC) of 0.77.

**Figure 6:**
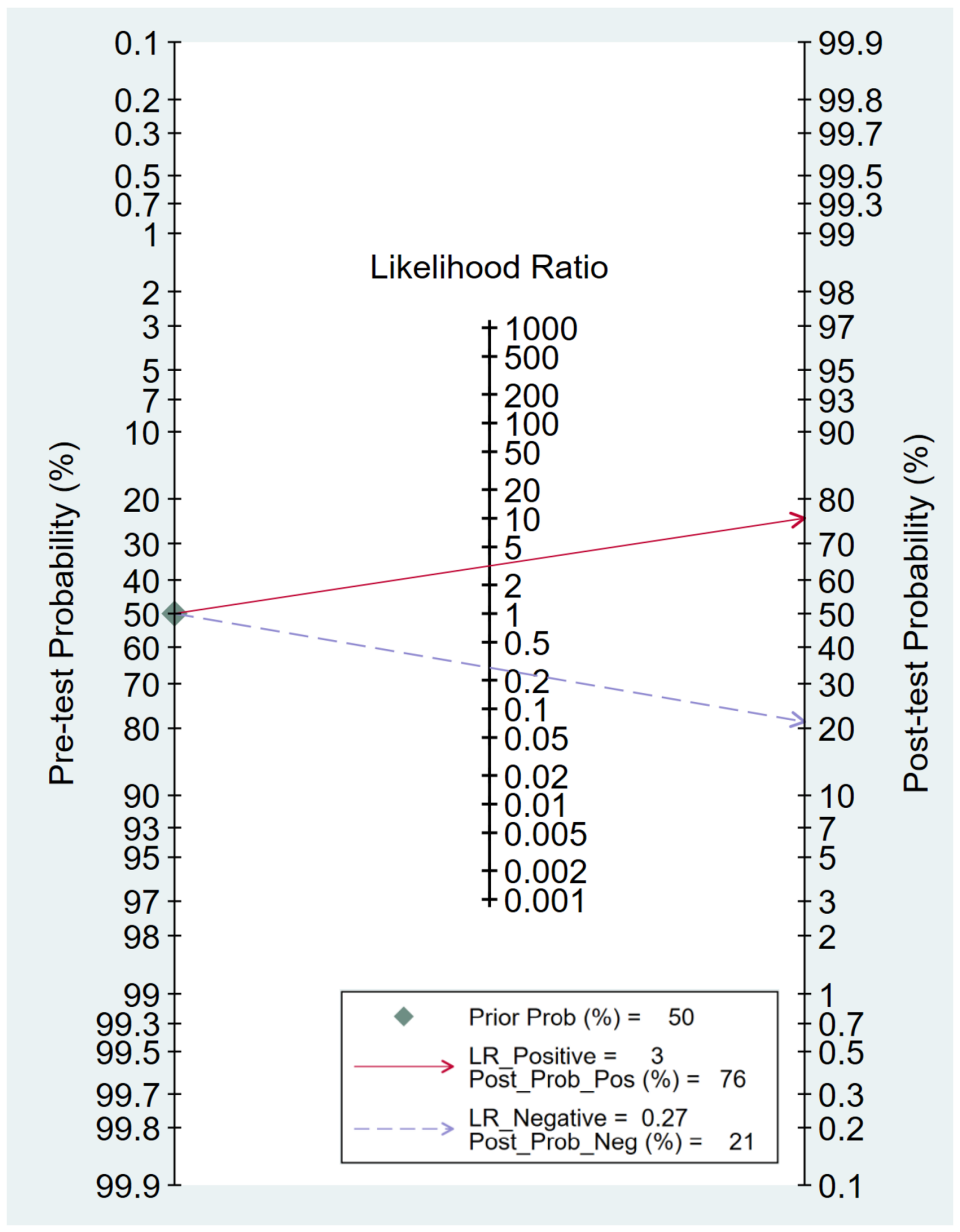
Fagan’s nomogram revealing the post-test probability of mild cognitive impairment (MCI)/mild dementia.

**Figure 7:**
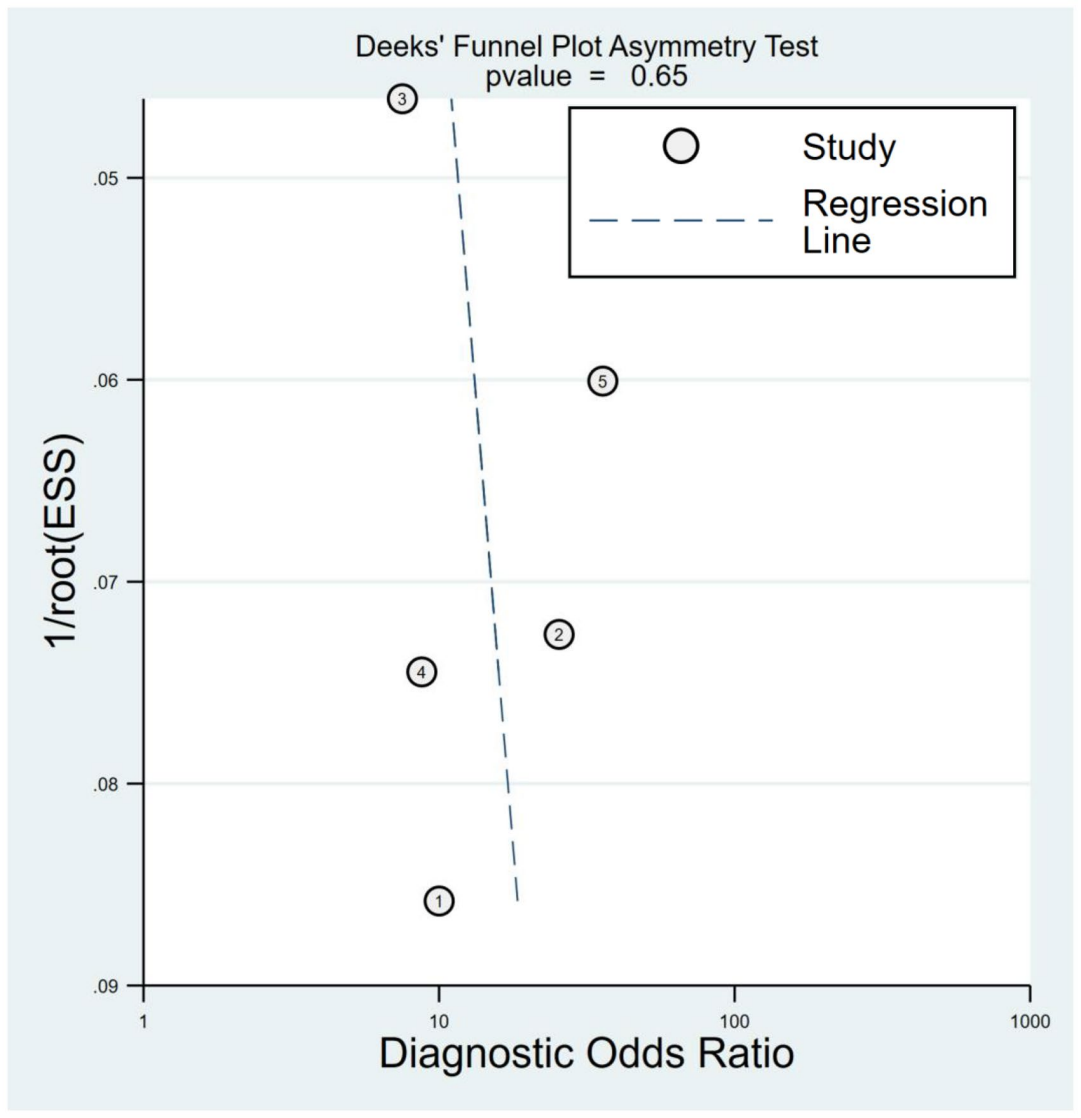
Deek’s funnel plot showing a low risk of publication bias (*p* = 0.65).

The combined sensitivity was 80% (68%–88%), whereas the pooled specificity was 75% (68%–80%) (Figure 4). The combined AUC was 0.77 (95% CI, 0.73–0.81) (Figure 5). The pooled positive likelihood ratio was 3 and the negative likelihood ratio was 0.27. If the pre-test probability was set at 50%, the post-test probability increased to 76% for a positive test and decreased to 21% for a negative test according to Fagan’s nomogram (Figure 6). The risk of publication bias was low (*p* = 0.65) (Figure 7). These results suggest that VACT is useful in diagnosing MCI/mild dementia.

## 4. Discussion

The increased use of brief cognitive assessments in primary care could improve diagnosis rates and provide early and appropriate treatment to patients. Considering the increasing attention toward cognitive impairment screening tools, to the best of our knowledge, this is the first meta-analysis designed to assess the diagnostic efficacy of the VCAT in identifying cognitive impairment, specifically including both MCI and mild dementia. Based on a meta-analysis of 1,446 patients from studies conducted in Asian countries (four in Singapore and one in Malaysia), patients with MCI/mild dementia had lower VCAT scores than those without MCI/mild dementia, with a mean difference of −6.85. VCAT showed a combined sensitivity and specificity of 80% and 75%, respectively, with an overall AU-ROC of 0.77.

In the current meta-analysis, VCAT showed a combined sensitivity and specificity of 80% and 75%, respectively, with an overall AU-ROC of 0.77. This finding indicates an acceptable diagnostic performance of the VCAT in distinguishing MCI/mild dementia from the healthy control group. In a previous systematic review, the AU-ROC for the MoCA and MMSE was 0.883 and 0.78, respectively (23). While these findings suggest that the MoCA and MMSE exhibit higher diagnostic efficacy than the VCAT, this comparison was not conducted head-to-head owing to the absence of primary data. Several studies have reported similar diagnostic efficacies for VCAT and MoCA (15, 21). Lim et al. (15) reported that the AU-ROC for detecting patients with MCI/mild dementia was 0.905 and 0.916 for the VCAT and MoCA, respectively, suggesting that both tests are highly effective in identifying MCI/mild dementia. Similarly, Ng et al. (21) demonstrated no statistically significant difference in diagnostic efficacy between MoCA and VCAT. Recent evidence from 13 studies has shown that the effectiveness of the MoCA in identifying MCI varies, with sensitivity and specificity ranging from 73.5% to 83.8% and from 70.8% to 91.3%, respectively, depending on the selected cutoff score (24). Considering that the VCAT demonstrated a combined sensitivity and specificity of 80% and 75%, respectively, both tools appeared to be effective in detecting MCI/mild dementia.

Traditional cognitive tests, including the MMSE and MoCA, have demonstrated limitations when applied to diverse populations, primarily owing to sociodemographic factors and linguistic dependency-related biases (24–28). In contrast, VCAT presents a more universally accessible alternative. For instance, a multicenter study across four Asian countries demonstrated that the language used to administer VCAT did not significantly affect its performance (15). Another study showed that even when adjusting VCAT scores for factors including race and education, the tool remained effective in differentiating between cognitively normal individuals and those with MCI from those with mild dementia (20). Furthermore, racial analysis of VCAT scores showed no significant disparities, emphasizing cross-cultural fairness (15). Contrary to language-dependent tests such as the MMSE and MoCA, which necessitate translation and cultural adaptation (29–31), the VCAT relies on visual cognition and nonverbal responses, thereby eliminating the need for such modifications. This feature makes it adaptable across diverse linguistic and cultural contexts. Moreover, traditional screening tools (e.g., MoCA) are known to exhibit a ceiling effect, wherein a large number of cognitively normal individuals score near the test’s maximum, thereby reducing the tool’s sensitivity in detecting MCIs (31, 32). Conversely, the VCAT shows a more normally distributed scoring pattern among cognitively normal adults without excessive clustering at the upper end. This absence of a ceiling effect more effectively enhances the ability of the VCAT to distinguish between normal cognitive function and MCI/mild dementia than its MMSE and MoCA counterparts (12, 33).

The emphasis of the VCAT on evaluating episodic memory and executive function, with half of the test specifically geared toward assessing episodic memory, makes it a potential screening tool for early AD detection and monitoring. A previous study indicated that deficits in episodic memory can signal the future development of Alzheimer’s dementia in otherwise healthy adults (34). Furthermore, another study established a correlation between VCAT scores and the presence of amyloid beta, a biomarker of Alzheimer’s pathology (35). Based on imaging findings, another study reported a possible link between VCAT scores and medial temporal lobe atrophy, a sign of neurodegeneration (22). Collectively, these findings suggest that VCAT is sensitive to cognitive domains and biological markers associated with the early stages of Alzheimer’s pathology. This makes the VCAT a promising clinical instrument for identifying early neurodegenerative alterations in individuals at the preclinical stage of AD.

In the studies included in the current meta-analysis, Low et al. (20) examined the VCAT scores derived from healthy comparisons and MCI/mild dementia groups, discovering significant differences in the results of memory-related tasks between these two groups. This observation was further supported by Soo et al. (22), who found that only memory-related domains exhibited pronounced differences between healthy comparisons and MCI/mild dementia groups (22). Pearson’s correlation analysis further revealed a high correlation between VCAT scores and memory domain neuropsychological assessments (22). Studies by Ng et al. (21) and Lim et al. (15), also yielded similar results, showing significant differences in VCAT scores across various domains between healthy comparisons and MCI/mild dementia groups, with memory exhibiting the most pronounced decline. These findings not only reflect the substantial weighting of memory tasks within the VCAT, but also underscores the effectiveness of the test in identifying MCI/mild dementia. A systematic review recommended the MoCA over the MMSE as the preferred tool for screening MCI/mild dementia in primary care settings (36). The finding that Ng et al. (21) revealed a high correlation (i.e., *r* = 0.819) between the MoCA and VCAT, further highlighting the robustness of the VCAT as a cognitive assessment tool.

Several studies have highlighted the feasibility of integrating the VCAT into clinical settings, reporting its ease of completion as a significant advantage (22, 37). The average time needed to complete the VCAT was comparable to that of the MoCA for both healthy controls (10.0 vs. 10.4 min) and those with cognitive impairment (15.4 vs. 15.4 min), indicating that participants typically perceived its instructions as easy or easier to understand than those of the MoCA (37). In a study by Soo et al. (22), 94.3% of the participants believed that VCAT instructions were either equivalent to or simpler than those of the MoCA. However, VCAT is designed for patients with normal visual capabilities, making it less suitable for those with visual impairments. To optimize MCI/mild dementia detection using the VCAT, we recommend its use in conjunction with a brief clinical interview. This should include checks for subjective memory complaints using tools such as the subjective memory complaints questionnaire (38) and an assessment for functional decline using measures such as the instrumental activities of daily living scale (39).

The use of a visual-based format in the VCAT not only minimizes potential variables that could influence the results but also facilitates digital implementation (14). This clinical application is particularly significant for individuals with undiagnosed dementia who do not actively seek medical care or consultation (40). When cognitive decline becomes a concern, digital technology makes it easier to conduct initial assessments at home by shifting away from old-fashioned paper-and-pencil methods to more efficient digital platforms (41). Moreover, growing acceptance of technology among older adults has spurred the development of remote digital cognitive assessments capable of detecting subtle cognitive shifts (42). Previous visual-based tests, such as the phototest for dementia, were largely developed using Western populations (43). However, the VCAT was conceived within Eastern cohorts, providing that our study added significance in confirming its diagnostic effectiveness, particularly within the Asian context.

The notable heterogeneity in our meta-analysis can be partially attributed to the study by Soo et al. (22), which reported lower sensitivity and AUC values than other studies. This discrepancy could arise from the unique composition of the study population, which consisted solely of patients with MCI (22). In contrast, other studies have included patients with both MCI and mild dementia. There is a distinction between MCI and mild dementia. While individuals with MCI show subtle but observable cognitive issues, those with mild dementia have more severe cognitive impairments that affect daily activities. The notable heterogeneity suggests that the VCAT might have reduced sensitivity in detecting MCI compared to mild dementia, which could explain the diminished sensitivity observed in Soo’s et al. (22) study. While analyzing data from patients with MCI or mild dementia can potentially confound the discriminative value of the tool, our results offer clinicians initial insight into the strengths and weaknesses of the test. We believe that future research may build upon our findings and further delineate the diagnostic value of VACT in specific patient populations.

The current meta-analysis has several limitations. First, all included studies were conducted in Asian populations, primarily Chinese participants in Singapore and Malaysia. This limits the generalizability of the findings to non-Asian and diverse populations. Further studies on other ethnicities and cultural groups are required. Second, only five studies were included in the meta-analysis. A greater number of high-quality studies would allow for a more robust quantitative synthesis and subgroup analyses to explore potential sources of heterogeneity. Third, the criteria used for defining MCI/mild dementia varied somewhat across studies, which could have introduced inconsistencies. Standardized diagnostic criteria improve the comparability. Fourth, data on key demographic variables, including gender, education, and language, have been inconsistently reported across studies. The impact of these factors on the VCAT accuracy could not be fully evaluated. Fifth, no data have been reported regarding the reliability, learning effects, or responsiveness of the VCAT to repeat administration. This further demonstrates its utility in monitoring cognitive changes over time. Finally, given the significant heterogeneity observed in the pooled sensitivity (94.23%), a systematic review might be better suited than a meta-analysis to present the existing literature on the use of VCAT in diagnosing cognitive decline. While pooling data in a meta-analysis can amplify the risk of misinterpretation owing to such pronounced heterogeneity, our findings underscore the limitations of the included studies and highlight potential directions for future research.

## 5. Conclusion

This meta-analysis of five studies involving 1,446 older adults indicated that the VCAT, as a nonverbal and visual-based cognitive screening tool, has moderate diagnostic accuracy in distinguishing individuals with MCI/mild dementia from cognitively normal controls. The pooled sensitivity and specificity were 80% and 75%, respectively, with an AU-ROC of 0.77. Further rigorous studies are required to confirm its applicability and validity across different populations and settings.

## Data availability statement

The original contributions presented in the study are included in the article/Supplementary material, further inquiries can be directed to the corresponding author.

## Author contributions

J-HH: Data curation, Investigation, Writing – original draft, Writing – review & editing. C-CL: Investigation, Writing – original draft, Writing – review & editing. I-WC: Investigation, Visualization, Writing – original draft, Writing – review & editing. J-YW: Methodology, Validation, Writing – original draft, Writing – review & editing. P-YH: Data curation, Resources, Writing – original draft, Writing – review & editing. T-HL: Data curation, Methodology, Writing – original draft, Writing – review & editing. K-CH: Conceptualization, Supervision, Visualization, Writing – original draft, Writing – review & editing.
