# Supplementary material for: Efficacy of the visual cognitive assessment test for mild cognitive impairment/mild dementia diagnosis: a meta-analysis
Source: Front Public Health. 2023 Oct 31;11:1293710. doi: 10.3389/fpubh.2023.1293710 (PMC10644725; doi:10.3389/fpubh.2023.1293710)
Supplement: Supplementary file 1 [file Table_1.docx]

**Supplemental Table 1.** Limitations enumerated for each of the studies included in the meta-analysis

| Study | Limitation |
| --- | --- |
| Low 2020 | - Small sample size (n=471) - Neuropsychological assessments were conducted in the same session, possibly causing a learning effect - Included only Chinese, Malay, and Indian participants in Singapore |
| Lim 2018 | - Small sample size (n=284) - Included only Chinese, Malay, and Indian participants in Singapore and Malaysia - Absence of subjects with lower education levels (< 6 years) |
| Ng 2022 | - Small sample size (n=184) - Limited to Malaysian Chinese population - Relatively high education level (mean = 12.0 years) - Insufficient information regarding the test’s accuracy in relation to its severity |
| Kandiah 2016 | - Small sample size (n=206) - Limited to Singaporean Chinese population - The effects of education, employment status, and participant’s primary language on VCAT’s diagnostic performance were not evaluated |
| Soo 2023 | - Small sample size (n=301) - Unequal group sizes for cases and controls - Limited to Chinese participants in Singapore - Relatively high education years (mean=13.6 years) |
